# Designing Digital Mental Health Tools to Support the Needs of Black Adults in the United States: Qualitative Analysis

**DOI:** 10.2196/73279

**Published:** 2025-10-06

**Authors:** Sarah Alexandra Popowski, Jonah Meyerhoff, Olivia Marin Allen, Theresa Nguyen, Terika McCall, Aderonke Bamgbose Pederson, Madhu Reddy, David Mohr, Rachel Kornfield

**Affiliations:** 1 Department of Preventive Medicine, Feinberg School of Medicine, Northwestern University, 750 N. Lake Shore Dr., 10th Floor, Chicago, IL, 60611, United States, 1 312-503-6576; 2 Mental Health America, Alexandria, United States; 3 Department of Biostatistics, Division of Health Informatics, Yale School of Public Health, Yale University, New Haven, United States; 4 Department of Psychiatry, Massachusetts General Hospital, Harvard Medical School, Boston, MA, United States; 5 Department of Informatics, University of California, Irvine, Irvine, United States

**Keywords:** technology, mHealth, SMS text messaging, mental health services, depression, anxiety, African Americans, health equity, race, mobile health

## Abstract

**Background:**

Depression and anxiety are associated with excess morbidity and mortality, constituting a major health care challenge. The prevalence of these conditions is increasing. In the United States, the health-related burden of depression and anxiety may disproportionately affect Black adults, who face unique stressors impacting their mental health and barriers to accessing treatment, including but not limited to systemic racism, discrimination, underdiagnosis of common mental health concerns (ie, depression, anxiety), limited access to culturally sensitive care, and mental health stigma within and outside Black communities.

**Objective:**

This study aimed to explore the mental health experiences of nontreatment-seeking Black adults, and how these experiences relate to their needs and preferences for the design of digital mental health (DMH) tools through user-centered design methods.

**Methods:**

This study included 25 nontreatment-seeking Black adults (aged 18‐61 years) with experiences of depression or anxiety to share their perspectives on how DMH tools can meet their needs. Participants were recruited either through social media advertisements or depression and anxiety questionnaires. All participants engaged in an asynchronous online discussion group in which they discussed their past and current mental health experiences, distinct challenges faced by Black Americans, and perceptions of DMH tools, as well as how such tools can be tailored to meet their mental health needs. Participants also completed a technology probe in which they used an automated mental health self-management text messaging tool (Small Steps SMS; Audacious Software) for 18 days. They shared their perceptions of the tool and ideas for specific design improvements in the discussion group. A subset (n=6) completed follow-up interviews to elaborate on their online discussion group posts.

**Results:**

All participants reported significant mental health concerns and difficulty managing related symptoms. A majority of participants (22/25, 88%) expressed that racism and mental health stigma severely impacted their mental health and limited opportunities to discuss their experiences within and outside Black communities. They were interested in the use of DMH tools for mental health self-management and nearly all participants (23/25, 92%) endorsed text messaging as a convenient way to introduce techniques for coping with symptoms of depression and anxiety; however, some participants strongly advocated for additional design features that they believed would improve the program, including the integration of content that centers the experiences of Black individuals, creating nonjudgmental spaces for discussing mental health experiences, and linking formal mental health treatment resources for those who want them.

**Conclusions:**

These findings suggest that our participants hold generally favorable views toward DMH tools, which can provide psychoeducation, self-management support tailored to the needs of Black adults, and a safe environment to address mental health concerns. Furthermore, it is critical to consider the role of racial discrimination and mental health stigma when designing inclusive and culturally sensitive DMH tools.

## Introduction

The United States is facing a burgeoning mental health crisis, in which depression and anxiety are at the forefront. Depression and anxiety are pervasive mental health conditions that affect 8.3% and 19.1% of adults each year, respectively [1,2]. They are often chronic and debilitating, with adverse downstream effects on education, employment, and quality of life [1,2]. The clinical presentation of depression and anxiety varies from person to person and between groups [3,4]. Symptoms of these conditions are common in Black adults and occur at a similar rate to that observed in White adults [5]; however, Black adults’ experiences of depression and anxiety are more likely to be chronic and severe [3,6]. Notably, in the United States, Black individuals’ mental health challenges are compounded by experiences of structural racism, inequity, discrimination, and bias in society at large, as well as in health systems [7,8]. Black adults report higher numbers of chronic and acute stressors compared to White adults, including adverse childhood experiences, racial microaggressions, and both interpersonal and institutional maltreatment [9-11]. Furthermore, research has found that stressful life events are associated with poorer mental health outcomes and an elevated risk of depression, anxiety, and suicide, as well as higher overall severity and chronicity of mental health problems [11,12]. Previous research also suggests that the effects of institutional racism across multiple domains of social determinants of health (ie, economic stability, educational access and quality, health care access and quality, neighborhood and built environment, and social and community context) increase the likelihood of psychological distress and negative mental health outcomes [13].

Symptoms of depression and anxiety are less likely to be treated in Black people in the United States [14], who may be deterred from seeking treatment by costs and other structural barriers like the limited accessibility of services in their neighborhoods, limited mental health literacy, and attitudinal barriers such as stigma and beliefs that treatment is ineffective [15]. Cultural factors in Black communities also have the potential to heighten barriers to formal help-seeking. These include the belief that mental illness is a weakness or moral failing [16,17], as well as preferring faith-based management of mental health [18]. Estimates suggest that Black adults are less than half as likely to access mental health services compared to White adults [19].

Black adults who do seek and receive formal care may find that existing treatments fall short in addressing their needs. When given the choice, many Black individuals express a preference for providers who are the same race as them (ie, patient-provider race concordance) [20]; however, underrepresentation of providers of color has made it difficult for most Black adults to achieve such concordance [21,22], which may lead to challenges building rapport with their provider [23] and less satisfaction with treatment [24]. Furthermore, available treatments may overlook culturally relevant aspects of mental health, such as stigma, spirituality, and racial identity [20,25]. Many Black adults may therefore be looking for ways to manage mental health concerns outside of the formal care system.

Recent efforts have included consideration of digital mental health (DMH) tools to address unmet needs, bridge gaps in care, and provide culturally sensitive mental health services. For instance, studies have sought to understand Black adults’ preferences for the design of chatbots to support managing chronic conditions [26], voice assistants to support older Black adults in seeking online health information [27,28], and DMH tools to better support Black women in the perinatal period [29]. However, limited work has focused on the needs of Black Americans in the context of scalable tools like automated text messaging programs for mental health self-management.

Text messaging is a particularly accessible method for delivering mental health support [30]. In the United States, cellphone ownership is almost universal, and high rates of text messaging extend across racial and ethnic groups and socioeconomic factors [31,32]. Furthermore, since text messaging is a built-in tool on the mobile phone, and among the most frequently used communication methods, it has extremely high reach and low barriers to entry [33]. Access can extend to individuals who may not own modern smartphones or reliably access a data plan [30]. Text messaging is also cost-effective in terms of program development, maintenance, and deployment [30]. Thus, it may constitute an appropriate modality for equitable, accessible DMH tools. Previous research suggests that Black adults send and receive text messages frequently and may be more likely to use text messaging overall compared to White adults [32]. Black adults also find text messaging to be a suitable approach for treating various health concerns [34].

Previous studies examining the use of text messaging tools to support health improvement across a wide range of medical conditions, including those targeting both specific diagnoses (eg, diabetes, hypertension, and asthma) and health-related behaviors (eg, medication adherence, smoking cessation, and weight loss), indicate positive effects on health outcomes [30]. The potential of text messaging has also been explored in mental health interventions targeting conditions such as depression, anxiety, suicidal thoughts and behaviors, and other mental health challenges, with studies demonstrating moderate yet significant positive effects. Thus, text messaging may offer an effective and scalable modality for delivering mental health support to individuals who otherwise may not receive care, although such programs also have possible risks and ethical considerations in the context of mental health treatment [35-37]. For example, concerns may include privacy and the risk of intrusiveness, management of sensitive health information, and the clinical appropriateness of automated responses to acute distress or crisis situations [35,38].

To address gaps in both the literature and the unmet needs of individuals facing barriers to traditional care, this study applies user-centered design methods to examine how Black adults who are not engaged in formal mental health treatment envision using a text messaging intervention to manage depression and anxiety symptoms. We recruited Black adults in the United States who self-reported experiences of depression or anxiety and sought—through an online discussion group and parallel text messaging-based technology probe—to deepen our understanding of their perspectives related to mental health support and how DMH tools might help to meet their needs. We posed the following two research questions: (1) What types of mental health challenges are faced by Black adults who are not engaged in formal treatment? and (2) How should digital tools deliver support to users to address their challenges, particularly in the context of automated text messaging? Answering these questions can help align scalable DMH tools to the needs of Black people in the United States, with potential for broad impact in addressing their unmet mental health needs and advancing mental health care equity.

## Methods

### Overview

This section describes how we worked with Black adult participants to understand their needs and preferences for DMH tools.

### Participants

Recruitment procedures for this research were fully remote, providing individuals across the United States the option to participate in the study from any convenient location and using their own devices. Participants were recruited through social media advertising (Instagram and Facebook) and through free online self-screening surveys for depression and anxiety—that is, the 9-item patient health questionnaire (PHQ-9) [39], or 7-item Generalized Anxiety Disorder scale (GAD-7) [40]—hosted by Mental Health America, a large nonprofit mental health advocacy organization. The advertisement text read, “You are invited to participate in a Northwestern online research project on Black and African American mental health,” followed by a link displayed in their social media feed or alongside their screening results. Interested individuals then were guided to a primary screening survey to assess study eligibility, with inclusion criteria specifying that participants should (1) identify as Black or African American, including individuals who also identify as mixed race; (2) self-report a history of depression or anxiety symptoms (whether or not they were professionally diagnosed); (3) reside in the United States; (4) be 18 years of age or older (or 19 years of age or older in Nebraska, reflecting the state’s age of majority); (5) have English language abilities sufficient to read, understand, and participate in study procedures; and (6) own and be willing to use a personal mobile phone. Individuals were excluded if they were currently seeing a therapist, counselor, or psychologist, or taking medications for their mental health symptoms. Individuals were also excluded if they reported a serious mental illness (eg, bipolar disorder and schizophrenia); if they had visual, voice, hearing, or motor impairments that would prevent completion of study procedures; or if they reported suicidal ideation with a current plan and intent to act. Furthermore, as the study involves receiving automated text messages, we confirmed that individuals provided the study team with a valid US mobile phone number before enrollment. Individuals who were deemed eligible after completing the primary screening survey were invited to complete a secondary screening survey, which evaluated the presence of suicidal thoughts, plan, and intent. Participants with a suicide plan or intent received risk assessment and resources and were not eligible to participate. Interested and eligible individuals were asked to sign a code of conduct stating participants would remain respectful to one another and refrain from sharing personally identifiable information (eg, name and address) within any of their responses. They were then asked to provide informed consent and were sent instructions to join the discussion group.

Consistent with guidance for research using asynchronous remote communities [41], we sought to recruit a small group size such that participants could participate actively and build rapport with one another. Of the 27 individuals who met eligibility criteria, provided consent, and enrolled in the discussion group, 2 did not respond to any prompts in the group and were not sent automated text messages or invited for follow-up interviews. Like recruitment procedures, all study activities were fully remote and allowed participants to engage from any convenient location using their own devices. Furthermore, study activities, apart from follow-up interviews, were available to participants asynchronously and could be completed at their leisure within the study timeframe. Data were collected between June 9, 2023, and July 27, 2023. The first discussion group prompt was posted on June 9, 2023, and participants were given the opportunity to engage in discussion group activities until July 3, 2023; however, some participants were interviewed through July 27, 2023, due to scheduling availability. Demographic characteristics for the 25 participants included are represented in Table 1.

**Table 1.** Demographics, symptom severity, recruitment source, academic achievement, and treatment history for US-based Black adults included in this qualitative analysis.

Characteristic		Discussion group (n=25)	Interview^a^ (n=6)
Age (years), mean (SD)		34.76 (10.96)	27.17 (10.59)
Depression or anxiety symptoms, mean (SD)			
PHQ-9^b^		9.96 (5.70)	10.33 (6.02)
GAD-7^c^		10.36 (5.63)	13 (3.95)
Sex, n (%)			
Female		20 (80)	6 (100)
Male		5 (20)	0 (0)
Race, n (%)			
Black or African American		25 (100)	6 (100)
More than one race^d^		1 (4)	0 (0)
Recruitment source, n (%)			
Mental Health America		3 (12)	0 (0)
Social media		22 (88)	6 (100)
Highest level of education, n (%)			
Some high school or less		1 (4)	1 (17)
High school graduate		5 (20)	1 (17)
Some college		11 (44)	2 (33)
Associate’s degree		4 (16)	1 (17)
Bachelor’s degree		2 (8)	1 (17)
Post-bachelor’s education		2 (8)	0 (0)
Mental health treatment history, n (%)			
Has seen a mental health professional		10 (40)	2 (33)
Previously prescribed medication for a mental health condition		7 (28)	0 (0)

a Interviewees were a subset of those who participated in the discussion group.

b PHQ-9: 9-item Patient Health Questionnaire.

c GAD-7: 7-item Generalized Anxiety Disorder scale.

d Participants who identify as Black or African American in addition to another race.

### Procedure

#### Asynchronous Online Discussion Group

To increase comfort discussing issues related to racial identity and mental health, participants were informed that all other study participants identified as Black or African American and had experiences of depression or anxiety. Participants were asked to create a pseudonymous account on the study platform [42]. The platform was programmed to release a new prompt every 3 days for 24 days (8 total prompts). Prompts centered on understanding participants’ mental health needs and management strategies, how these are impacted by racial identity, their perceptions and experiences of technologies as a method of mental health support, and ideas for how automated text messaging tools could be adapted to meet their needs. Each prompt posed a series of questions related to a specific topic (see Multimedia Appendix 1).

Participants were asked to provide substantive responses, defined as contributions that advance the discussion such as sharing personal stories or opinions, posing clarifying questions, or reflecting on others’ posts. No minimum or maximum word count was imposed on participant responses. Participants were compensated based on the number of prompts to which they responded. They could earn additional compensation by replying to at least one other participant’s comment on each prompt. Text-based responses were automatically recorded by the study platform [42] and downloaded at the study’s end.

#### Text Messaging Technology Probe

Participants were enrolled in the Small Steps SMS program partway through the study (on day 6), allowing them the opportunity to engage in broader discussions of DMH tools and Black mental health before focusing on their experiences with and perceptions of the text messaging program. The program delivered support for self-management of depression and anxiety symptoms via daily interactive dialogs [43,44]. The content of the messages was drawn from 11 evidence-based psychotherapy strategies and included a variety of formats such as psychoeducation messages that introduce each strategy and how it works, peer stories, opportunities to write or receive supportive messages, and skill-building exercises (see Multimedia Appendix 2). The peer stories included in the program were collected from a previous study that used group discussions and writing prompts to gather peer support narratives. Materials were subsequently edited and curated by research staff before being included; thus, participants were not involved in live exchanges with peers. Messages varied in length, reflecting the program’s structure, which incorporated both brief, responsive interactions and more comprehensive psychoeducational messages. For example, some texts consisted of only a few words such as “Good morning” or “Thank you for sharing” and were included primarily to enhance the natural conversational tone of the program. In contrast, other messages were considerably longer, often consisting of several sentences and were intended to deliver valuable psychoeducational content. The number of messages sent each day varied based on participants’ interactions with the program; participants who responded more frequently may have received a greater number of messages (eg, follow-up questions). Participants could engage with Small Steps SMS as much or as little as they wanted. They could also elect to end all messages by sending “STOP,” although no participants did so. Participants were asked to reflect on their experiences with and impressions of Small Steps SMS in the discussion group.

#### Interviews

Participants were invited to complete optional one-on-one semistructured interviews, lasting about 20‐25 minutes, at the end of discussion group activities. Interviews gave participants the opportunity to voice ideas or thoughts they did not share in the discussion group and provide additional feedback on Small Steps SMS, as well as ideas for DMH tools more broadly. In total, 6 participants completed interviews. All interviews were conducted via the Zoom teleconferencing program by a member of our research team. Participants were able to turn their video on or off based on their preferences. With participants’ permission, interviews were audio recorded and transcribed.

### Ethical Considerations

Study activities were approved by the Institutional Review Board (IRB) at Northwestern University (STU00211168). Informed consent was collected from all participants prior to enrollment in the study. Participants were compensated US $7 for responding to each online discussion group prompt and US $2 for replying to at least one other participant’s response to each prompt, for a total possible compensation of US $72 for all discussion group activities. Participants who completed interviews were compensated an additional US $8. Our research team includes mental health clinicians and researchers, human-computer interaction researchers, and research staff. Our team includes individuals who are and are not Black, whereas all participants were Black and most were women. To enhance rapport and comfort based on a foundation of shared identity (ie, race and sex), all interviews were conducted by a Black female team member. Clinical psychologist team members provided input throughout the study to ensure participants’ wellbeing and safety, which included advising on the study design and code of conduct, developing risk management protocols, and providing supervision of research staff in monitoring screening data, monitoring discussion posts for potential indicators of suicidal thoughts, behaviors, or nonsuicidal self-injury, and conducting risk assessments when necessary. All individuals who completed the screening process or participated in the study were given a list of resources for accessing 24/7 mental health support, if needed (eg, suicide prevention hotline and crisis text line). The website focusgroupit.com [42] was selected to host discussion group activities based on its security features and user-friendly design. The platform adheres to privacy regulations like General Data Protection Regulation and the EU-US Privacy Shield and does not sell user data. To further protect participant confidentiality, no personally identifiable information was stored on the focusgroupit.com website [42]; participants were identified through randomly generated codes linked to their study ID and were asked not to share identifying information. The discussion group was accessible only to enrolled participants. Privacy protocols were reviewed to ensure compliance with IRB requirements. Responses in the discussion group were monitored daily for compliance with the code of conduct. Across all activities, research staff had a risk management protocol in place, such that participants’ sharing of any information signaling risk to themselves or others would prompt the research team to contact the participant and administer risk assessments and provide resources as needed. No such risks emerged, and therefore, no follow-up was conducted.

### Data Analysis

Discussion group and interview data were subject to thematic analysis using an inductive, semantic approach, which allowed themes to emerge naturally from the data with a focus on the explicit meanings conveyed in participants’ words [45]. A total of 3 coders, who are authors of this paper, first became immersed in the data by reading discussion group and interview transcripts. They performed open coding to identify preliminary codes and then met to discuss and prioritize these codes, guided by assessment of each code’s relevance to the research questions. Prioritized codes and their definitions were captured in a shared codebook. The coders then applied the shared codebook to overlapping transcripts using the qualitative data analysis software Dedoose (SocioCultural Research Consultants) and subsequently met to discuss and resolve coding discrepancies and to refine and consolidate the codebook. After 4 overlapping coding rounds, discussions ceased to yield codebook revisions, at which point the coders divided the uncoded transcripts and applied the final codebook. Key themes were identified that encompassed the coded data, which were discussed with the rest of the research team for feedback. Excerpts were selected to illustrate each theme.

## Results

### Overview

All of the Black adults in our study reported facing profound challenges in managing their mental health concerns and were receptive to using technology to help overcome these challenges. In the following sections, we describe the primary themes, which draw on responses from both the discussion group and follow-up interviews. Interview participants were a subset of discussion group participants, and their responses expanded on key points that emerged in the group discussion. First, we describe the unique mental health challenges participants faced related to navigating discrimination and stigma and perceptions of limited opportunities to share their mental health experiences with others due to concerns about racial bias. Next, we describe participants’ receptivity to an automated text messaging program that allows them to address their mental health concerns independently through a convenient medium. Finally, we describe how participants envision that digital tools can be designed to meet the unique mental health needs of Black adults through integrating culturally appropriate content written by and for Black individuals, creating safe spaces for disclosure of mental health concerns, and supporting help-seeking beyond the DMH tool among interested users.

### Mental Health Challenges of Black Adults

Black adults in our study overwhelmingly reported being distressed by their mental health symptoms, which included a lack of motivation, fatigue, reduced pleasure in daily activities, loneliness, a sense of overwhelm, and anxiety, among others. As the sections below describe, participants’ everyday experiences of depression and anxiety were compounded by habitual negative experiences that relate to race, particularly experiences of discrimination, stigma, and invalidation.

#### The Role of Racism and Discrimination in Mental Health

When asked how mental health issues differ for Black individuals than for other groups, 88% (22/25) of participants shared that they face pervasive exposure to discrimination and racism across multiple domains of life, which negatively impact their mental wellbeing. A participant referred to these experiences as “anti-Blackness,” sharing:

Black people unfortunately have to deal with everyday anti-Blackness and racism that exacerbates existing mental health issues.

[Participant 5, Discussion Group]

Participant 5 provided additional insight into the damaging effects of anti-Blackness, emphasizing that its harm can extend beyond direct targets through vicarious racism, such as when accounts of anti-Blackness are shared among Black community members or broadcast publicly on media channels.

If we are not encountering it in our day to day lives personally, then we are inundated with instances of racism in the news and social media.

[Participant 5, Discussion Group]

Participants described several specific effects that anti-Blackness had on their mental health, including increasing anxiety and decreasing motivation and energy level. Participant 6 felt that repeated instances of discrimination were linked to their anxiety, relaying that, “We deal with discrimination and injustices and fear and it affects our mental health because we are more prone to be on edge and anxious about certain things and situations” (Participant 6, Discussion Group). Another participant echoed this sentiment and provided further context, noting that encounters with authority figures, particularly law enforcement, can heighten anxiety and fear: “You have to always be on guard. When you’re out in public you hope that you don’t have any interactions with the police because that could be the end of your life” (Participant 10, Discussion Group). Concerns that interactions with law enforcement could reinforce experiences of discrimination and negatively impact mental health were shared by other participants, including Participant 12: “...we go through discrimination, blacks get arrested quicker than any other race. It does effect your mental health” (Participant 12, Discussion Group). Others connected anti-Blackness to “burn out” and withdrawal, such as Participant 25:

For most of us, we are surrounded by whiteness in school, work, and even socially, but there is a level of masking that can lead to feeling burned out, and then social anxiety about re-entering those spaces can pop up. I remember reading about Black workers dreading the return to the office due to the amount of microaggressions they had avoided while working from home.

[Participant 25, Discussion Group]

Participant 25’s account highlights the extent to which anti-Blackness pervades participants’ lives and identifies schools and workplaces as common settings for microaggressions. Participants emphasized the work that goes into navigating White spaces, observing that this continuous effort could exhaust them and hinder their success in both school and the workplace.

Participants further explained that the feeling of being inundated with anti-Blackness can also impact one’s self-concept and self-worth. Participant 5 described:

I feel like sometimes being bombarded with images and videos of Black people being killed at the hands of police and other forms of state sanctioned violence, even the election of a white supremacist president to the highest office in the land, it can really feel as though my life doesn't matter, even though it does absolutely.

[Participant 5, Interview]

In this statement, Participant 5 linked exposure to what they described as “state-sanctioned violence” and feelings of worthlessness.

Participant 7 agreed, stating, “It’s just like what did we do so wrong for people to hate us so much? Being treated like that makes a lot of people feel unworthy & they may want to harm themselves” (Participant 7, Discussion Group).

Their experiences suggest that feelings of worthlessness can contribute to self-harm ideation.

#### Disclosure and the Risk of Invalidation

Most participants (14/25, 56%) emphasized the importance of disclosure as a means of addressing experiences of anti-Blackness and processing the resulting impacts on their own mental health, as well as the broader mental health of the Black community. For instance, Participant 15 shared, “open and honest conversation can bring upon a wealth of relief” (Participant 15, Discussion Group), suggesting that disclosure might aid in reducing mental health symptoms. Although most participants expressed interest in mental health disclosure, they perceived limited opportunities to discuss their experiences, particularly those related to anti-Blackness. In fact, 72% (18/25) of participants mentioned being wary to discuss these issues. They reported hesitation to discuss their experiences with Black and non-Black individuals alike, including health care providers. Participant 14 summarized this sentiment: *“*A lot of us really don’t like talking to therapists, family or friends. So, we keep it bottled on the inside and try to deal with it our self” (Participant 14, Discussion Group). Participants overwhelmingly avoided disclosing how racism impacted their mental health and wellbeing to non-Black individuals, primarily citing fears of invalidation. Participant 5 described:

Dealing with discriminatory behavior can be so exhausting and depressing, not to mention the added frustration having our experiences of racism be downplayed or outright dismissed by non-Black people.

[Participant 5, Discussion Group]

This comment suggests that a dismissive response to one’s disclosure can have a compounding effect on the initial instance of discrimination. Another participant feared that disclosure could activate damaging stereotypes, relaying that:

In my personal experience, it was and is still sometimes hard for me to be vocal without being labeled as the “angry black woman.” I've been working on not dimming my own light because of someone’s ignorance.

[Participant 1, Discussion Group]

Participants also noted that mental health disclosure could be perceived as a sign of weakness, with one person sharing, “Feel weak when we express our feelings” (Participant 2, Discussion Group). Another participant agreed with this sentiment, stating: “I agree, we express our feelings, but yet no one listens” (Participant 13, Discussion Group). Despite wanting to share their experiences broadly with confidants of other races, participants were attuned to potential negative repercussions that discouraged and often prevented disclosure.

Many participants (14/25, 56%) mentioned that therapy could hypothetically create a supportive environment for disclosing and processing their experiences. Yet, concerns about invalidation extended to mental health care providers. For example, Participant 18 reported:

It is hard for [therapy] to work, because not everyone can understand the feelings black people feel unless they experienced it themselves. It is important that the feelings of Black individuals are not invalidated.

[Participant 18, Discussion Group]

A preference for Black providers was discussed by 8 participants (32%). Participant 15 thought that having shared experience would allow them to disclose more candidly to a provider, describing that, “It makes it easier to be understood without shame or saying something that might be offensive to a non-black individual” (Participant 15, Discussion Group). Some also described that providers should understand the history of racial injustice in the United States. Participant 25 stated:

I think support systems must be well-versed in the cultural and historical foundation that plays out in our daily life. I think individual, as well as group therapy, led by those who have the training and cultural background, is a great start.

[Participant 25, Discussion Group]

While many participants thought they could benefit from working with providers who could understand their experiences, they expressed some skepticism that such providers would be available to them.

Finally, participants’ concerns about disclosure often extended to other Black individuals, particularly when sharing details of their mental health issues, and 64% (16/25) mentioned that their mental health had been negatively impacted by stigma within the Black community. They feared that sharing their struggles with family, friends, and other community members could lead to judgment or ostracism. Participant 21 explained:

I feel like there’s this stigma within the African American community, especially with surrounding mental health. I know I’ve been told by family members—not immediate family members, but kind of distant family members like, “Oh, therapy is not for our people; our type of people don't go to therapy.” We don't broadcast our problems; we kinda internalize it.

[Participant 21, Interview]

A similar sentiment was relayed by Participant 15, who shared:

I have loved ones who think that talking about depression means I need to be placed in a mental facility. It is sad because some of them unknowingly need to seek therapy but most likely will not because they fear the stigma behind it.

[Participant 15, Discussion Group]

Some participants connected negative attitudes about mental health treatment to religious beliefs. Participant 11 stated:

African Americans deal with shame, and backlash for having depression and or anxiety. I grew up with a Christian mother and grandmother that tells you to pray about everything. Mental illness in general in the black community is a taboo thing.

[Participant 11, Discussion Group]

Participants expressed optimism that attitudes toward mental health treatment were shifting such that Black communities were becoming more accepting; however, Participant 25 reflected on both progress and disappointment, expressing pride in the growing openness around mental health within the Black community while simultaneously mourning the lack of support for mental health concerns historically:

I am proud of the evolution our community has experienced when it comes to [mental health], but I also feel saddened for all of us who suffered without support, and were forced to just “pray” it away.

[Participant 25, Discussion Group]

Overall, participants faced a dilemma. They felt a strong desire to have their experiences acknowledged and understood by others but perceived few appropriate outlets within which to share, leading many to keep their mental health struggles private. As we describe in the next section, DMH tools were largely perceived positively since they provided a low-burden, private, and nonjudgmental method for receiving day-to-day support.

### Receptivity Toward an Automated Text Messaging Program

Although participants in our study were not seeking formal mental health care, they largely saw the value of self-guided tools to help them manage their mental health privately, particularly when tools took advantage of convenient communication channels.

#### Introducing and Normalizing Self-Management

After they began receiving messages from Small Steps SMS in the technology probe, participants described the program positively, with one person suggesting that it aided in motivation, “I like that it keeps me motivated throughout the day” (Participant 8, Discussion Group). This perspective was reinforced by a second participant who responded, “Yes I feel the same way. It keeps me motivated and on track” (Participant 20, Discussion Group). Participant 16 believed the program empowered them to become more proactive in their own mental health. They described:

I think this program would be very beneficial for African Americans like myself. Especially when we are not ready to talk to someone face to face … I would definitely sign up for the program to help me cope with my anxiety and depression.

[Participant 16, Discussion Group]

Indeed, some participants reported they tried out the strategies supported by Small Steps SMS, such as Participant 15, who emphasized their success in challenging negative thoughts (one of the strategies the program supports). They described:

But reading those different tips and the different self-care things that were said, how to take that negative thought and kind of turn it into a positive one, that’s been very helpful.

[Participant 15, Interview]

Some other strategies highlighted by participants included cognitive reappraisal, “I like receiving the messages. They have really given me things to think about in a different perspective” (Participant 4, Discussion Group), and relaxation techniques, “Also [Small Steps messages] help me deal my angry issues and help me calm me down. The meditation is really good. Just help you relax too” (Participant 22, Discussion Group). Participants also appreciated that the program felt more comfortable and approachable than face-to-face treatment, particularly because it was perceived to be private and judgment-free while allowing participants to feel anonymous, with Participant 16 describing:

I really enjoy getting the text messages. It really helps when I'm down and depressed and keeps my mind off things for that moment … it’s like having your own personal counselor no judgment at all that makes me feel comfortable and to open up about my feelings more.

[Participant 16, Discussion Group]

For some participants, the nonjudgmental nature of the program may have fostered a sense of normalization that allowed them to be more accepting of and open about their mental health challenges. This normalization may have been achieved, in part, through the user-generated content integrated throughout Small Steps SMS in the form of peer stories and support messages. Participant 18 described:

Usually I don't like reading long messages, but if it was a story about how [a person] overcame something and the lessons that they learned from it, I actually loved reading those because it felt really personal and like something I could relate to

[Participant 18, Interview]

Similarly, Participant 5 reported:

I was not expecting to receive instances of people’s personal experiences, and it was honestly my favorite part of the experience. I think having people share their experiences makes me feel less alone

[Participant 5, Discussion Group]

Although Small Steps SMS was automated, peer stories could provide a sense of relatability and social support that was otherwise lacking and were endorsed by 40% (10/25) of participants as a strongly favored feature.

#### Convenience of Text Messaging

Participants liked that digital tools like Small Steps SMS were convenient, addressing several barriers to traditional mental health treatment, including time and cost. Participant 15 reflected that:

This program is convenient as it does allow someone the comfort of seeking help without the hassle of an in-person visit. Programs like this one are time and money saving.

[Participant 15, Discussion Group]

The system was also perceived to be convenient since it operated via text messaging, a function of the mobile phone that participants all routinely used, and because messages were brief and required little active engagement. For example, Participant 24 described:

Well, I guess one thing for me in particular, since I'm very much a workaholic, was the reminders of little self-care things I thought was nice. And just the reminders to kinda take some time to just decompress, so to speak.

[Participant 24, Interview]

At the same time, participants appreciated that the system allowed 2-way communication. This was described by Participant 11, who shared: “I liked the fact that some of the messages were interactive and required some participation” (Participant 11, Discussion Group). A few participants mentioned potential challenges with receiving mental health support via text messages, including the frustration of receiving too many messages in one day, or too often, and finding them too lengthy. However, participants overall endorsed the low burden of receiving push messages and also recognized that asking the user for input and participation could add value.

### Tailoring Digital Mental Health Tools for Black Adults

Participants described several ways the program, and other DMH tools, could be adapted to help them navigate the specific mental health challenges that emerge for Black adults.

#### Adapting Content to Reflect Black Individuals’ Experiences

Most participants (15/25, 60%) stated that Small Steps SMS should directly address the unique challenges faced by Black adults with mental health concerns. Participant 15 described, “The program should list some common topics black people face on a daily basis such as racism, various traumas, and other systematic barriers” (Participant 15, Discussion Group). Participants also endorsed educating users on the mental health effects of racism. Participant 5 shared:

I think having topics such as systemic racism and the adverse effects on the mental health of Black Americans could be very beneficial since it serves to validate the daily injustices we face due to attitudinal racism

[Participant 5, Discussion Group]

In addition to facilitating understanding of bias and discrimination, participants wanted the program to provide actionable guidance for navigating these challenges. Participant 7 suggested, “support should help just by letting us know that everyone isn’t racist or mean and teach us how to properly cope and deal with these things” (Participant 7, Discussion Group). Similarly, Participant 19 thought that the program should help connect users with resources around a range of issues faced by Black individuals:

Well racism and injustice is still alive and kicking. Social issues, high unemployment rates, policing problems … we deal with a lot … maybe provide information for different avenues to assist with resolving problems

[Participant 19, Discussion Group]

Participants felt that content addressing these issues should reflect Black voices. Participant 15 described, “anything that can be shared from someone that identifies as the same race with me, that’s positive” (Participant 15, Interview). Similarly, Participant 17 stated, “especially if the content sent was authored and curated by Black Voices with Blackness in mind” (Participant 17, Discussion Group). Some participants expressed a particular interest in non-professional content written by other Black Americans. Participant 11 shared,

I would like to see relatable stories that highlight being African American, the stigma of mental health treatment/diagnosis in our community, juggling parenting, working, and on top of that battling mental health. The stories should vary in outcomes, successful and failed outcomes, [relatable] uplifting stories, funny stories, inspirational stories.

[Participant 11, Discussion Group]

Several participants also endorsed integrating quotes from famous Black individuals, including “well known people, like Oprah” (Participant 25, Discussion Group). Similarly, Participant 18 shared:

Sending inspirational quotes by famous Black people I think would help. Because they will see people who are like them who have been in situations that are stressful and have made it through the situations. So that would be encouraging.

[Participant 18, Discussion Group]

Participants saw value in integrating diverse Black voices into the content of a messaging program, including professionals, peers, and celebrities.

#### Constructing Safe Spaces for Social Sharing

Although the version of Small Steps SMS used in our technology probe featured user-generated content composed by other individuals with mental health concerns, some participants thought that the system should go farther in how it supports social interaction.

In fact, 36% (9/25) of participants suggested facilitating direct conversation between Black users of a DMH tool, often drawing inspiration from the discussion group through which we conducted the study, which allowed them to compose long-form messages and engage with one another while maintaining anonymity. These features led to a sense of safety, as Participant 16 described: “I definitely appreciate that I can be open and honest about my feelings without negative judgment. [This discussion group] has been very beneficial for me and my mental health” (Participant 16, Discussion Group). Participant 15 agreed, stating:

This group is nice, and I, too, would consider this a safe space. I feel that I can be open and honest and will get back the same in return. I do not feel worried about being judged, I can be completely who I am, and still feel valued.

[Participant 15, Discussion Group]

This comment suggests that a safe space is one where all participants can be uninhibited in expressing themselves, without fear of judgment.

A few participants (12%, 3/25) spoke to the importance of establishing a sense of safety by ensuring that forums were accessible only to Black individuals with mental health concerns. For example, Participant 25 suggested “making sure there is gatekeeping to protect the space, so that it’s only for people who are Black American (or Biracial) and with a history of mental wellness challenges” (Participant 25, Discussion Group). Participant 23 shared this view, explaining that gatekeeping is essential given the scarcity of outlets for people like them: “I feel like it should be only catered to black Americans with mental issues because we don’t have a lot of safe spaces to talk about these things at all” (Participant 23, Discussion Group). However, a participant who identified as multiracial relayed past experiences in which they had felt excluded from Black spaces and expressed that the program should also include adults with mixed racial backgrounds. They stated:

I think it'd be nice if you kind of opened it up to, maybe not people who were just fully African American, but that also identify as African American, but something else as well, because I feel like oftentimes, people try to take that away from people.

[Participant 21, Interview]

Participants also discussed how creating a safe environment for disclosure would require moderation, or the active involvement of individuals whose job is to establish and uphold community guidelines. Participants once again took inspiration from the study’s discussion group, which was moderated by the study staff. Participant 17 described, “I think being a highly moderated and regulated platform allows people to speak about their mental health candidly while maintaining anonymity” (Participant 17, Discussion Group).

Finally, a sense of safety was one in which sharing was optional and could happen over time as participants became comfortable. A considerable number of participants (40%, 10/25) described that reading about others’ experiences was valuable regardless of their own desire to share, providing a sense of reassurance and connection, or an understanding that they were not alone. Participant 9 reported, “I appreciate this group. It showed me I’m not losing my mind that there are other women going through the depression, anxiety etc” (Participant 9, Discussion Group).

Ultimately, participants expressed that feeling safe and supported in their environment would make them more likely to open up about their mental health experiences, with 24% (6/25) of participants mentioning more favorable attitudes toward disclosure following the conclusion of the focus group. Furthermore, they drew from their positive experiences in the discussion group to highlight the value of interaction, boundaries, and moderation, all of which allowed them to feel they could share freely, without the risk of invalidation that characterized many other spaces they inhabited in their day-to-day lives.

#### Connecting to Other Sources of Support

While participants in this study were not involved in formal care, many saw the potential value in it, and this increased for some over the course of the study. Participants described that a program like Small Steps SMS may initially appeal to them because of its focus on privately managing one’s concerns, but that over time it might inspire individuals to seek additional formal or informal mental health support. For example, Participant 3 reported, “I think this opened my mind to possibly seeking mental health counseling” (Participant 3, Discussion Group). Similarly, Participant 5 described, “when I read stories of people reaching out to their pastors or their therapists, or a trusted friend, it just reminded me that I could seek out someone in my own circle” (Participant 5, Discussion Group).

Recognizing this possible evolution in attitudes around treatment, 32% (8/25) of participants mentioned that they wanted a DMH program to help users locate Black mental health care providers, who were perceived to be equipped to provide culturally sensitive support. For example, Participant 24 reported:

I think it would be great if the system had an option to see resources for POC [people of color] in their area. Maybe team up with black therapists on this as well. Another idea, would be the option to chat with a black therapist [real person] if necessary or if the AI alone isn't tailored to the individual enough

[Participant 24, Discussion Group]

Participants agreed that such features must be optional but saw value to integrating resources within a DMH tool to allow users flexibility in how to continue their mental health journeys.

Some participants also indicated that using the Small Steps SMS program and participating in the discussion group could inspire greater disclosure of their mental health challenges to important people in their lives. To support these behaviors, participants suggested that a DMH tool could provide guidance for navigating mental health stigma within Black communities. Participant 18 reflected that:

It is hard for [Black people] to accept that we need help. I think this program will give many people the push they need to get help or even talk to family or friends. especially if it introduces people to resources that helpful for mental health

[Participant 18, Discussion Group]

Thus, while many participants were initially receptive to Small Steps SMS because it could be used privately, a few (3/25, 12%) suggested that the program should support transitions in how individuals cared for their mental health, including greater willingness to engage in formal and informal help-seeking.

## Discussion

### Principal Findings

Our goal in this study was to understand the challenges faced by Black adults in the United States and to clarify their preferences and perspectives for the design of DMH tools that can be used outside formal care, including automated text messaging tools. Our research was guided by the following two research questions: (1) What types of mental health challenges are faced by Black adults who are not engaged in formal treatment? (2) How should digital tools deliver support to users to address their challenges, particularly in the context of automated text messaging? Our findings suggest that participants perceived anti-Black racism to affect their mental health daily, and that these experiences translated into a deep need for disclosure and social validation that they could not easily satisfy in their daily lives. They deemed automated text messaging and DMH interventions more broadly to be an acceptable approach to addressing mental health concerns through the introduction of mental health self-management strategies; however, they also clearly identified ways to better represent their experiences within the content of the Small Steps SMS program, as well as complementing pre-scripted content with safe opportunities for social sharing and pathways to access mental health support beyond the DMH tool. Below, we discuss the implications of our findings for understanding the mental health challenges of Black Americans and for designing DMH tools for this population.

### Understanding Mental Health Challenges and Disclosure Needs of Black People

Our study suggests that participants made many connections between incidents of anti-Black racism and their affective states, thoughts, and behaviors, including linking exposures to racism to changes in their mood, anxiety level, productivity, and feelings of devaluation. They also emphasized that these effects are cumulative, reducing educational or career achievement and self-esteem and potentially leading to burnout or even suicide or self-harm-related thoughts and behaviors.

Participants in our study reported that they were affected not only by firsthand experiences of discrimination, but also by exposure to others’ experiences as conveyed through social networking apps and mainstream news. Exposure to state-sanctioned violence was especially upsetting. The implication of this finding is significant given the highly publicized nature of events like the murder of George Floyd in 2020, with video footage of the murder disseminated widely both through news media and social media. These findings underscore a need to better understand the effects of vicarious experiences of anti-Black racism, including how these effects may be shaped by the type of event (eg, institutional discrimination, violence, and microaggressions), the channels through which experiences are shared (eg, news media, social media, or face-to-face), and accompanying commentary and contextualization. Additional research should examine strategies to mitigate the harms of vicarious trauma without compromising opportunities to access the benefits of sharing, such as building public understanding, spurring collective action, or coping with personal distress.

Findings from our study suggest that mental health disclosure, particularly in relation to instances of anti-Black racism, was viewed by participants as a pathway to coping. Conversely, inhibition was perceived as unhealthy, leading participants to feel the pressure of their “bottled up” distress. This view of disclosure as a potential solution for intense negative emotions is widely held in US culture [46,47]. In a model from clinical psychology, disclosure is compared to a fever as both a sign of an underlying destructive process and a restorative force [46]. There is some evidence for such views; Pennebaker, in his “expressive writing” paradigm, proposed that the act of writing about troubling thoughts and feelings could spur various benefits for writers, even if what they wrote was never shared with others [48]. The benefits of private writing may include relieving stress, facilitating the development of new perspectives, coping with illnesses and traumas, or improving general health and well-being [49]. However, the effectiveness of disclosure as a coping strategy, as well as its associated health benefits, may largely depend on cognitive processing of stressors, which plays a crucial role in race-based trauma recovery and adjustment [50,51]. In parallel, a body of literature has accumulated on the unique benefits that can come from social sharing of emotions, which may include eliciting social support, discovering alternative perspectives, prompting reciprocal disclosure, and potentially deepening relationships [52-54]. Despite its potential benefits, social sharing is complicated when disclosing identities or experiences that are stigmatized [55,56]. Concerns about how one’s disclosure will be received are common, and anticipating negative reactions can lead to selective disclosure or nondisclosure [57,58], which is consistent with our findings.

Some work has examined how complexities of disclosure increase for individuals who have multiple stigmatized identities [59-61], reflecting that systems of oppression related to each identity can interact with one another to transform and sometimes magnify the experience of bias [62]. As described in our findings, participants found that identifying as a Black person with mental health concerns could compound the burden of racism and mental health stigma and reduce disclosure opportunities. Participants greatly desired to discuss the role racial discrimination played in their day-to-day mental health and well-being, but they were subject to judgment from non-Black individuals when sharing about anti-Blackness, and they anticipated stigma from Black individuals when sharing about their mental health struggles. The resulting unmet need for disclosure provides an important backdrop for introducing DMH tools for Black people.

### Design Considerations for DMH Tools Catering to Black Americans

Our findings can help to inspire design and refinement of DMH tools to better match the priorities and needs of Black people in the United States. Below, we discuss opportunities to deliver self-management support and culturally tailored content through text messaging, to construct safe digital spaces for social sharing, and to provide pathways to other forms of care.

#### Representing Black Experiences and Voices in Text Messaging Programs

These findings are generally encouraging regarding automated text messaging programs for Black people in the United States. Following our technology probe, participants reported positive views of automated text messaging as a modality for mental health support. The text messaging program was recognized for its capacity to support private and convenient self-management, which allowed them to bypass formal disclosure or help-seeking. In a previous study investigating the acceptability of text messaging for facilitating one-on-one mental health support among Black women, participants voiced privacy and confidentiality concerns [63]; however, these concerns were not independently raised by our participants, who were encouraged to voice any potential concerns about DMH tools and text message-based tools like Small Steps, although they were not asked directly about privacy issues. The reason for this difference is unknown. It is possible that an automated program is less likely to raise concerns about social judgment or misrepresentation of identity relative to one where individuals discuss their mental health with professionals. Participants in our study also identified as nontreatment-seeking, expressing interest in alternative methods of mental health support (eg, automated mental health support) rather than traditional approaches involving trained professionals. Thus, participants may have been inclined to accept DMH tools and to perceive automated support more favorably. In addition, participants received messages from Small Steps in the context of a research study where they had learned about the program through recruitment and orientation procedures, as well as discussing DMH tools with peers in the discussion group, which could have contributed to reduced concerns about the modality of text messaging. Future research should explore how privacy concerns may be thoughtfully addressed in texting programs, or other DMH programs, that are tailored to the needs of Black adults [64,65].

Many of our participants’ concrete suggestions for adapting the text messaging program centered on the content. Participants advocated for representing issues related to race and elevating the voices of Black people. Suggested topics included racism and discrimination, stigma, and daily life stressors affecting Black communities (eg, unemployment and policing). Participants wanted the program to be clearly designed “for us” (ie, Black people with mental health concerns). A primary avenue to achieve this is by including Black voices. Efforts may extend to didactic content (eg, written by Black mental health professionals) and stories of peers managing similar challenges. Although the stories in our text messaging probe were not designed to represent the issues of Black people in particular, our findings suggest they were relatable based on shared mental health challenges. Relatability could likely be further enhanced by including stories explicitly written by Black Americans. These content-related suggestions are feasible to implement within a text-based program, including through crowdsourcing methods [66], or potentially soliciting stories directly from users of the DMH tool as part of their therapeutic disclosure process [64].

#### Integrating “Safe Spaces” Within DMH Tools

While some design ideas focused on content that could be integrated within the existing structure of Small Steps SMS, others pushed the boundaries of what an automated text messaging program can deliver. Although the discussion group was originally intended as a space for participants to share feedback on Small Steps SMS, participants unexpectedly drew on the experience as a model for future DMH tool design, emphasizing the importance of community and peer connection. In particular, they highlighted features that could create a “safe space” for sharing without the risk of judgment or invalidation. In other studies, asynchronous discussion groups have been used not only to establish understanding of users but as delivery tools for social support [67]. Our findings suggest that these groups may provide an appealing approach to support Black individuals’ mental health. Participants indicated that the benefits of the discussion group stemmed from its interactive format, including threaded conversations, the group’s moderation processes, and the boundaries of group membership. Although less frequently highlighted by participants, the group also featured anonymity, researcher-constructed discussion prompts, a text-based format, and asynchronicity. In what Walther [68] termed “hyperpersonal communication,” asynchronicity can allow participants time to deliberately present themselves, while the reduction of nonverbal cues in text-based exchanges allows readers to focus on the content of the disclosures, potentially supporting idealization of one another and bonding that may even exceed what face-to-face communication allows.

There are various technologies that could potentially be applied to construct digital “safe spaces” for therapeutic disclosure. Our discussion group shared many similarities with online support groups, which individuals can use to connect with geographically dispersed strangers, and which are typically bounded by a shared experience or identity, creating a comfortable context for disclosure of sensitive issues [69]. Many online support groups include features like interactivity, anonymity or pseudonymity, and moderation [70,71]. Using SMS text messaging or other messaging apps could potentially allow for both interpersonal and programmatic support via the same technology. However, it is important to consider the specific context under which DMH tools facilitate the disclosure of distressing events, particularly traumas. Disclosure alone, if not accompanied by appropriate support and guidance, may increase emotional arousal, which has the potential to be harmful for individuals [50,72]. Thus, moderators may require specific training in responding to trauma disclosures and linking individuals to appropriate resources.

Regardless of the technology used, constructing supportive peer-to-peer communication environments is generally less scalable than delivering fully automated tools, particularly when forums are moderated and when boundaries are enforced around membership. In our study, participants’ eligibility was vetted, and staff were trained in a code of conduct, risk management protocols, and moderation procedures. They also reviewed, daily, all new messages posted within the group and acted, if needed, to uphold the code of conduct and ensure safety. Such human labor is often the most complex or costly aspect of a DMH program [73,74], although efforts are underway to make moderation processes more scalable, such as through crowdsourcing [75] or automation [76,77], although these efforts must be complemented by appropriate human-in-the-loop review [78,79]. While challenging, providing moderated social spaces may be essential to address the paucity of disclosure opportunities Black people experience and their specific fears of invalidating responses.

#### Extending Openness Beyond the DMH Tool

Despite not being involved in formal mental health care, many participants were open to the possibility of seeking it in the future. The primary barrier they reported was their limited ability to find providers with shared racial backgrounds. Many participants described that they would connect to formal mental health services that are designed with Black people in mind. They suggested that a DMH tool should increase understanding and availability of culturally appropriate services, including by providing directories of Black clinicians and connecting users to immediate technology-delivered care from Black providers (eg, teletherapy). Thus, participants challenged the division of support into formal and informal programs, suggesting that these should be integrated.

Furthermore, some participants found that using the text messaging program and engaging in the discussion group changed how they felt about treatment. Some described a more general change in openness, such that they considered sharing about their mental health with close others to whom they had not previously disclosed. Others noted that their engagement with Small Steps SMS helped them recognize gaps in their existing support systems, prompting them to seek additional or alternative forms of help. Designing for ongoing help-seeking and eventual termination would recognize the evolving nature of individuals’ support needs and the ways that different forms of informal, formal, and interpersonal support may be complementary over the long term.

### Comparison With Previous Work

Our findings add to a growing literature establishing the negative effects of pervasive racism, bias, and discrimination on the health of Black adults [7,80,81]. Past work has examined the effect of racism on outcomes like chronic stress, general mental and physical health, and cardiometabolic conditions [82,83]. For example, a study that used data from Gallup and the US Census before and after the 2020 death of George Floyd to examine anger, sadness, depression, and anxiety among Americans found that these emotions and symptoms spiked among Black people [84]. In addition, by leveraging mobile phone technologies, research has begun to examine the in-the-moment effects of experiences of discrimination, which may lead to longer-term outcomes [85,86].

Our work provides support for some established approaches to inclusive design of DMH tools. Past work suggests that story-based text messages (ie, narratives) can be a valuable form of mental health support and are well-received when they are perceived to be authentic, when they balance positivity with a realistic portrayal of the narrator’s struggles, and when narrators are relatable to the receiver [66]. The relatability of narratives has also been enhanced by closely matching the circumstances of the story authors and receivers, such as when African American veterans compose narratives of managing hypertension for other African American veterans with the same condition [87]. Inclusive design may also involve creating a “safe space” wherein users can directly interact with those with shared identities and express themselves authentically [88]. Some work has sought to support conversation with peers via texting, including as one-to-one text chats. Work by Ybarra et al [89] facilitated one-to-one chats between sexual minority adolescent boys alongside pre-scripted automated messages that supported practicing safe sex and HIV prevention. Moderated group chats have also been applied to improve psychosocial functioning [90], increase preventive care [91], and support outpatient psychiatric care [92].

Furthermore, past work has emphasized the need for technologies to support transformations that occur, over time, in individuals’ social needs as they use a DMH program. For example, individuals may move from needing informational support when considering and initiating behavior change to needing partnership and accountability when maintaining their change [93]. Our findings suggest a similar need for technologies to align with changes in openness to help-seeking. This may include framing help-seeking as an evolving process, demystifying it, and prompting periodic consideration of the sorts of help a person might want, as well as having external resources available to participants at any time. Decision-making tools may also help the user arrive at the appropriate treatment approach given their current priorities [94,95]. Such processes of deciding and committing to continue or leave treatment are built into some psychotherapies (ie, termination) [96], but largely have not been explored in relation to DMH tools. Such an approach might avoid a common problem in which users report that their disengagement with a tool, even if that tool has met their needs, becomes a source of guilt and stress that is counterproductive to their mental health [97].

### Limitations

This study has limitations. First, although we sought to minimize barriers to participation, our sample represents those willing to discuss mental health issues and interact with researchers. Given our research focuses on DMH tools, our sample may also represent those who are interested in or comfortable with technologies. In addition, our study included web-based and SMS components, which limits our ability to speak to the needs of those who lack access to a mobile phone or the internet. Our sample overrepresents women. This is typical in DMH research but limits our ability to represent the needs of Black men. Mental health stigma can be especially high for Black men [17], leading some to argue for programs specifically targeted or tailored to Black men [98]. Our study also included anyone reporting their race as Black or African American, therefore encompassing individuals from diverse ethnic and cultural backgrounds, including individuals who may be African American, Black Caribbean, or African migrants and immigrants, or second-generation immigrants, among others. However, we did not collect data on these backgrounds or how they shaped participants’ experiences and views. Future research should consider within-race ethnic differences when designing and tailoring DMH tools. Our inclusion criteria also allowed for multiracial participants (if they also identified as Black or African American), but their representation in our sample was low. The needs of multiracial individuals are important to consider, especially when tools are tailored toward a specific racial identity or background. In addition, our sample size limits the generalizability of our findings. Although this study encouraged participants to voice concerns regarding mental health support via text message, they were not explicitly prompted to reflect on specific issues like privacy concerns. Further research is necessary to understand how risks related to text messaging for health intervention are perceived by diverse populations with mental health concerns and to develop strategies that mitigate these risks while maximizing the benefits of digital tools.

### Conclusions

Despite the potential for DMH tools to help address racial disparities in mental health care, this has yet to be achieved. To contribute to the design of tools that can reach and benefit Black adults outside of formal care, this paper examined how nontreatment-engaged Black adults perceived an existing automated text messaging tool and how they envisioned DMH tools being designed and refined to better reflect their experiences and meet their needs. Participants shared that the mental health challenges of Black individuals differ from other racial groups in that they are compounded by inequity, discrimination, and bias, and that these issues must be directly addressed within mental health tools. Participants endorsed text messaging as a medium for delivering convenient and nonstigmatizing self-management support but also endorsed platforms that can facilitate moderated conversations between individuals with shared racial backgrounds, emphasizing that such opportunities are essential since they lack other “safe spaces” within which to discuss the role of race in their mental health. Thus, scalable DMH tools like Small Steps SMS can alleviate barriers to treatment that may disproportionately burden Black adults by providing a nonjudgmental space for mental health disclosure, which can help to ameliorate the extent to which experiences of racism, stigma, and bias limit Black adults’ access to care. Future work should consider how text messaging can support social interaction between Black participants, as well as consider other platforms that can construct a safe social environment to complement self-management programs.

## Supplementary material

10.2196/73279 Multimedia Appendix 1 Discussion group prompts analyzed in this study.

10.2196/73279 Multimedia Appendix 2 Example participant SMS text messaging interactions in this study.
