# Supplementary material for: Designing Digital Mental Health Tools to Support the Needs of Black Adults in the United States: Qualitative Analysis
Source: JMIR Form Res. 2025 Oct 6;9:e73279. doi: 10.2196/73279 (PMC12500225; doi:10.2196/73279)
Supplement: Multimedia Appendix 1 [file formative-v9-e73279-s001.docx]

**Discussion Prompt 1: Technologies**

To get started, let’s get to know each other by chatting about technologies we use every day and those that are our favorites. This could be things like emailing, texting, listening to podcasts, blogging, using social media, browsing the internet, watching videos or shows, or playing video games.

- What technologies are your favorite to use and why?
- How can technology, especially those that are your favorites, be used to help people feel less sad, down, empty, or anxious? Or, if you don’t think technology can be used to help people feel less sad, down, empty, or anxious, why do you think that is?

**Discussion Prompt 2: Feeling down, depressed, or empty**

It is very common to feel sad, depressed, empty, or anxious, but these feelings are different for everyone. Some mostly feel down. Others don’t feel like doing many things and don’t have their normal energy, some replay moments over and over in their head, some feel frustrated and stressed, some have problems at work and in their relationships, and most feel some combination of these things.

- Which of these thoughts, feelings, and experiences are the worst and/or the hardest on you?

To feel less sad or down, some people do activities like exercise, meditate, listen to music, color, cook, think about happy things in their lives, or talk to people they trust.

- What do you do to help feel less sad, depressed, or empty? How do these things help you when you feel sad, depressed, or empty?

**Discussion Prompt 3: Mental wellbeing for Black or African American people**

- Are there things that Black/African American individuals deal with that other groups might not have to deal with? How do you think that affects our mental health and well-being?
- How should support (like therapy, support groups, counseling, mental health apps and tools, etc.) help Black/African American people to address these issues? Are there types of support you can imagine would be helpful but that aren’t easily available?

**Discussion Prompt 4: Text Messaging**

We want to know how you feel about receiving text messages to help deal with sadness or depression, anxiety, or other mental health concerns. Please answer the following questions about your initial impressions of the Small Steps text messaging program.

- Did anything surprise you about the experience of receiving messages? If so, what?
- What did you like best about the experience of receiving messages, and why?
- Was there anything that disappointed you or didn’t meet your expectations about the experience of receiving messages, or that needs improvement?

**Discussion Prompt 5: Types of Messages**

You should now have been receiving messages for about 6 days. These messages have covered a variety of topics and have taken different forms. Some days, you might get background information on skills to help with sadness and anxiety (like gratitude, positive activities, or daily rhythms), and then a suggestion for how to try that skill out in real life. Some days, you might get stories from other people. Sometimes you might be asked to write messages for other people, or receive messages written by others.

- Based on your experiences so far, what types of messages have you found most interesting, and why? Which (if any) do you think might be most helpful to you in dealing with mental health concerns like sadness and anxiety?
- Were there any types of messages that didn’t work very well for you, or that you think we should change?

**Discussion Prompt 6: Making Small Steps Work for Black and African American People**

In an earlier session, we talked about how mental health issues might be different for people who identify as Black or African American, or how different sorts of support might be needed. This might mean that programs like Small Steps should work differently to meet your needs. We are curious what we can do to make this program engaging and helpful for people who identify as Black or African American.

- What would people who identify as Black or African American like about or be interested in about a program like Small Steps? What would be some common topics that would be interesting for people identifying as Black or African American?
- Are there changes we can make so that Small Steps would be more engaging or more helpful to people who identify as Black or African American? This might mean changing the way the program interacts with you or responds to you, adding new content, changing when messages are sent, or anything else.

**Discussion Prompt 7: Stories in Small Steps**

We have found that including stories written by real people in Small Steps is helpful to provide inspiration and a sense of connection to others.

- If we were to include stories by and for people who identify as Black or African American in Small Steps, what types of stories would be most helpful? What sorts of situations should they describe?
- Give an example of a story that might be helpful to others. The story might be about how you’ve taken a step to deal with feelings like being depressed, down, empty, or anxious. It’s ok if you didn’t completely resolve the problem; you can write about a time when you made some progress, or took a step that helped you feel a little better. You can write as though you were keeping a diary or writing to a close friend. Note: please do not use any full names or other identifying details in your stories. You can change the names of people or places you mention (e.g., "My friend John").

**Discussion Prompt 8: Editing Small Steps Messages**

You’ve now received a number of messages through the Small Steps program. We are curious how you might edit these messages to make them clearer, more interesting, more relevant, more helpful, or just to change the tone and wording. Below are some example messages you may have received.

Message 1: Good morning! Today we'll send messages about self-compassion. Self-compassion involves acting kindly and gently toward ourselves, even if we make mistakes or are feeling down.

Message 2: Good morning! Today we'll send some messages about daily routines. This is all about bringing consistency to things like meal times, bed times, socializing, and other daily activities. Stability in our routines can keep us grounded and free up our time and energy to focus on other things.

Message 3: Here's one thing you can do right now to practice gratitude: First, think of something you are grateful for. It doesn't need to be anything big. It can be a person, place, or thing. Second, once you have that thing in mind, just keep thinking about it for 30 seconds or so. Think about what you like about it and how it makes you feel. Just appreciate it.

- Pick one of the messages above (1, 2, or 3). Write a new version of the message, changing as little or as much as you want. Describe what you changed about the message, and why.
- Do you have any other thoughts about how we should edit the messages in this program, or other types of messages we should include?
