# Supplementary figures and images for: Designing Digital Mental Health Tools to Support the Needs of Black Adults in the United States: Qualitative Analysis
Source: JMIR Form Res. 2025 Oct 6;9:e73279. doi: 10.2196/73279 (PMC12500225; doi:10.2196/73279)

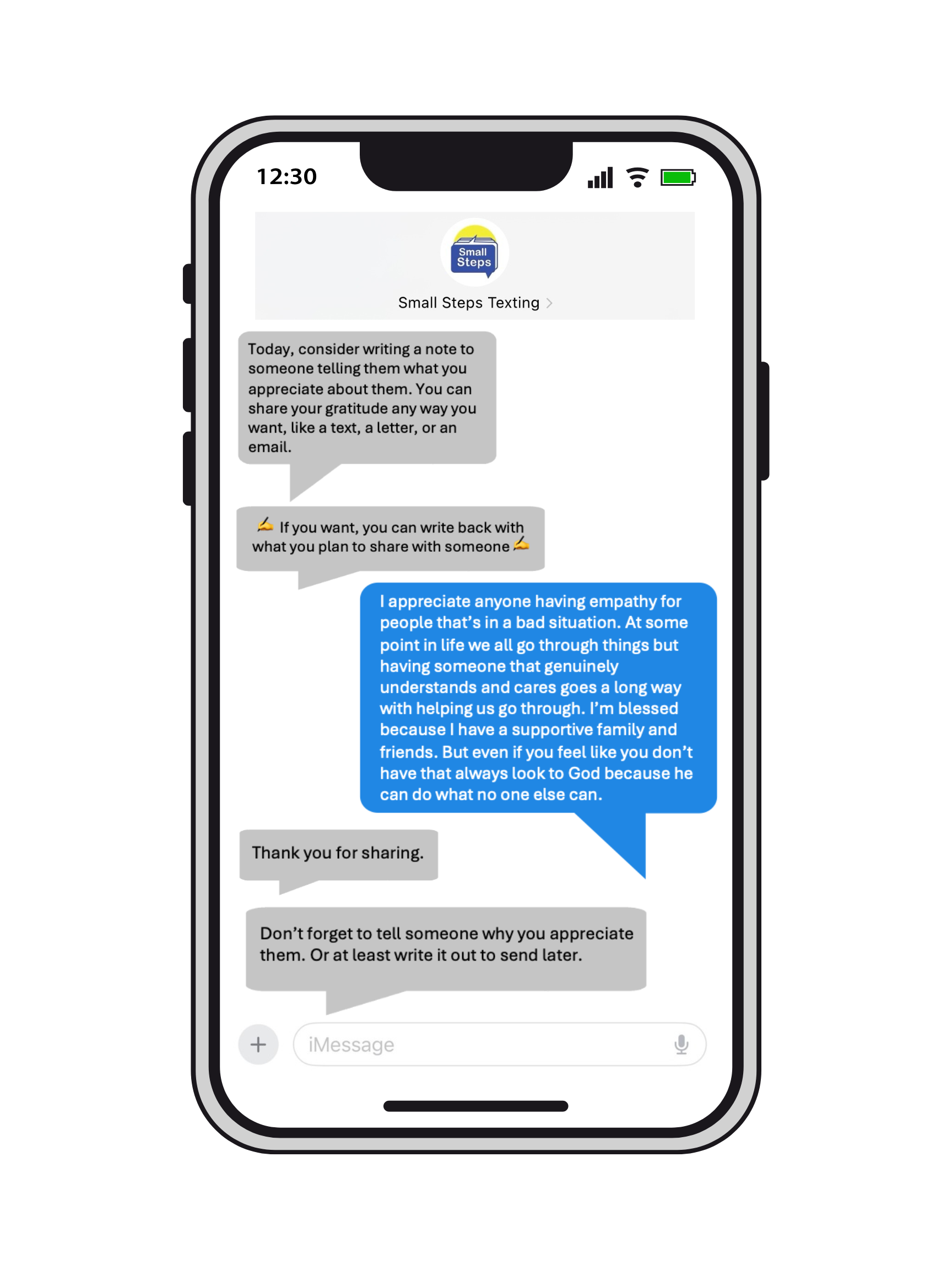


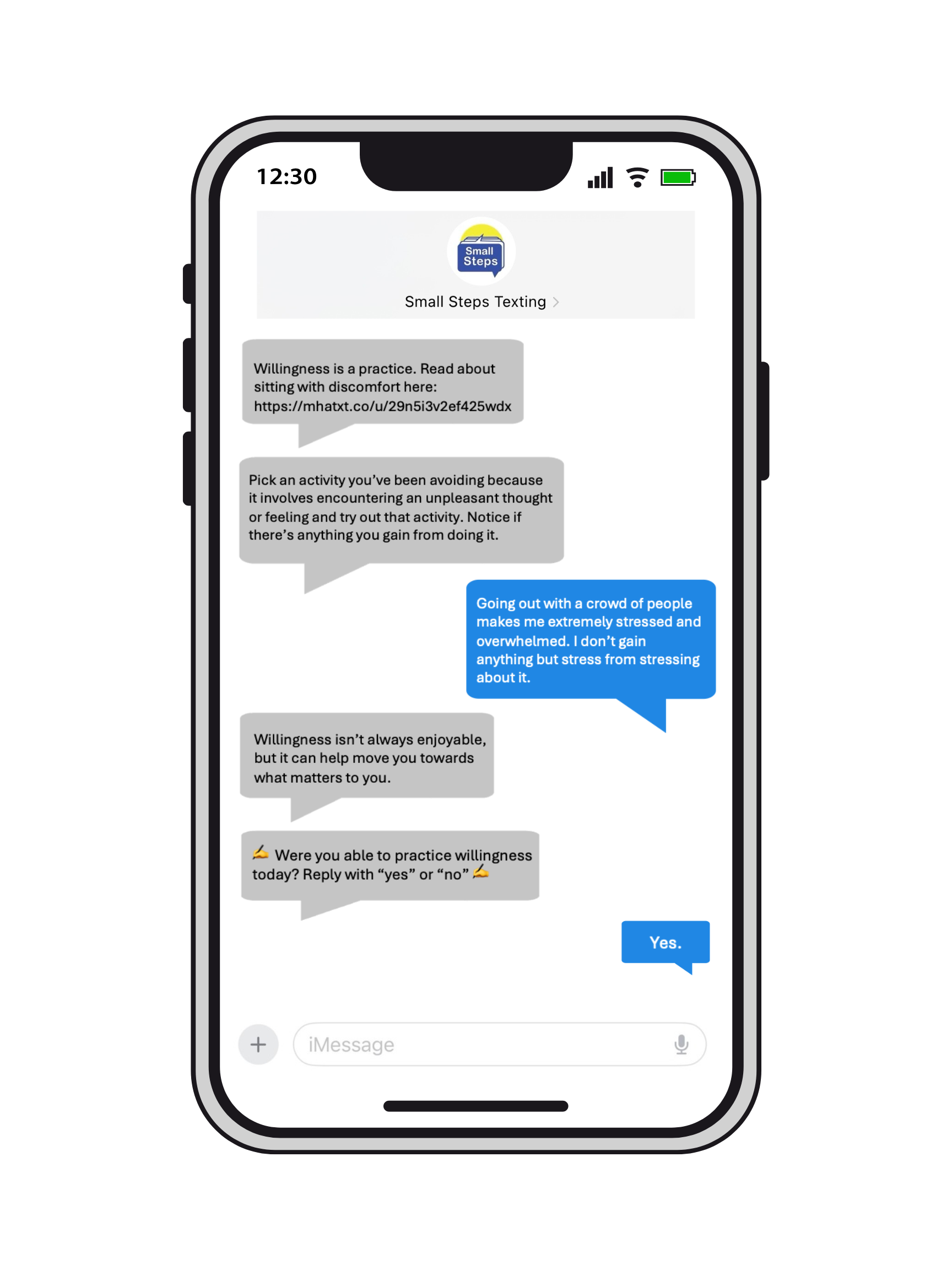

Supplement: Multimedia Appendix 2 [file formative-v9-e73279-s002.docx]
